# Targeted therapies in non–small cell lung cancer and their cardiovascular impact: mechanisms, clinical profiles, and strategies of management

**DOI:** 10.3389/fphar.2026.1790897

**Published:** 2026-03-19

**Authors:** Simone Nardin, Francesca Vezzoli, Gianluca Cognolato, Rocco Mollace, Beatrice Ramella Pollone, Federica Biello, Davide Cao, Marco Tagliamento, Matteo Sarocchi, Matteo Pagnesi, Monica Verdoia, Benedetta Conte, Salvatore Grisanti, Carlo Genova, Alessandra Gennari, Matteo Nardin

**Affiliations:** 1 Division of Medical Oncology, Maggiore University Hospital, Novara, Italy; 2 Department of Internal Medicine and Medical Sciences (DiMI), School of Medicine, University of Genova, Genova, Italy; 3 Department of Translational Medicine, University of Piemonte Orientale, Novara, Italy; 4 Cardiology Unit, Humanitas Gavazzeni, Bergamo, Italy; 5 Department of Experimental Medicine, University of Rome Tor Vergata, Rome, Italy; 6 U.O. Clinica di Oncologia Medica, IRCCS Azienda Ospedaliera Metropolitana, Genoa, Italy; 7 Department of Biomedical Sciences, Humanitas University, Pieve Emanuele, Milan, Italy; 8 Cardiovascular Disease Unit, IRCCS San Martino Policlinic Hospital, Genova, Italy; 9 Department of Medical and Surgical Specialties, Radiological Sciences and Public Health, Institute of Cardiology, ASST Spedali Civili, University of Brescia, Brescia, Italy; 10 Division of Cardiology, Nuovo Ospedale degli Infermi, ASL Biella, Biella, Italy; 11 Medical Oncology Unit, ASST Spedali Civili di Brescia, Department of Medical and Surgical Specialties, Radiological Sciences and Public Health, University of Brescia, Brescia, Italy; 12 Internal Medicine, Department of Medicine, ASST Spedali Civili di Brescia, Brescia, Italy

**Keywords:** cardiotoxicity, cardiovascular, cardiovascular diseae, lung cancer, NSCLC, targeted therapy

## Abstract

The therapeutic landscape of non–small cell lung cancer (NSCLC) has been profoundly transformed by the widespread adoption of molecular profiling and the development of targeted therapies, like tyrosine kinase inhibitors (TKIs), antibody–drug conjugates (ADCs), and bispecific antibodies (BsAbs). These agents have significantly improved survival and quality of life in molecularly selected subgroups, potentially converting NSCLC into a chronic disease requiring prolonged treatment exposure. However, extended survival has led to increasing recognition of cancer treatment–related cardiovascular (CV) disease as a clinically relevant and sometimes dose-limiting complication. Unlike conventional chemotherapy, CV toxicities associated with targeted therapies frequently arise from on-target or off-target interference with signaling pathways that are essential for myocardial survival, endothelial function, vascular regulation, and the cardiac conduction system. From common pathophysiological mechanisms, a broad spectrum of clinical manifestations arises, ranging from asymptomatic electrocardiographic changes to arterial hypertension, dyslipidemia, venous thromboembolism, arrhythmias, and heart failure. This review provides a comprehensive overview of CV toxicities associated with targeted therapies in NSCLC, integrating mechanistic insights with clinical evidence. We summarize class-specific CV risk profiles across EGFR, ALK/ROS1, RET, MET, NTRK, BRAF, and KRAS-G12C inhibitors, as well as ADCs and BsAbs, highlighting both shared and distinct patterns of cardiotoxicity. As targeted therapies continue to expand across disease stages and treatment lines, CV toxicity is expected to play an increasingly important role in therapeutic decision-making. Integrating CV considerations into oncologic care is therefore essential to preserve treatment continuity, optimize long-term outcomes, and maximize the benefits of modern targeted therapies in NSCLC.

## 1 Introduction

Non-small cell lung cancer (NSCLC) remains the leading cause of cancer-related mortality worldwide, accounting for approximately 85% of all lung cancer diagnoses and representing a major global health burden despite significant therapeutic advances in recent decades (Bray et al., 2024). The widespread adoption of molecular profiling has profoundly reshaped the therapeutic landscape of NSCLC, enabling the identification of actionable oncogenic drivers and the development of targeted therapies tailored to specific molecular alterations. Consequently, tyrosine kinase inhibitors (TKIs), antibody-drug conjugates (ADCs), and, more recently, bispecific antibodies (BsAbs) have become integral components of standard treatment algorithms across multiple lines of therapy (Carlisle et al., 2025; Huang et al., 2025; Syal et al., 2025). These therapies have significantly improved survival and quality of life in patients with NSCLC, frequently reshaping the disease into a chronic condition that requires prolonged or continuous treatment (Hernandez-Martinez et al., 2024; Peshin et al., 2025; Rothe et al., 2025).

However, extended survival has also led to an increased recognition of treatment-related toxicities, with cardiovascular (CV) adverse events emerging as clinically meaningful and, in some cases, dose-limiting or therapy-withdrawal complications. Unlike classical chemotherapy-induced cardiotoxicity, CV effects associated with targeted agents predominantly arise from on- or off-target modulation of signaling pathways that are critical not only for tumor progression but also for CV homeostasis, including endothelial integrity, myocardial metabolism, vascular tone, and cardiac electrophysiology (Lyon et al., 2022). In this context, cardio-oncology has emerged as a multidisciplinary field dedicated to preventing, detecting, and managing CV complications related to cancer therapies while ensuring optimal oncologic outcomes. Given the complex interplay between treatment-associated toxicities and pre-existing risk factors, the need for integrated CV care is increasingly evident (Camilli et al., 2025).

Despite growing awareness of these issues, CV toxicity in the context of targeted therapies for NSCLC remains incompletely characterized, and standardized monitoring and management strategies are not uniformly implemented in clinical practice. In particular, there is a need to integrate mechanistic insights with clinical evidence to develop tailored cardio-oncology approaches that account for the unique pharmacological properties of different drug classes, including TKIs, ADCs, and BsAbs. Immune-related cardiotoxicity has been widely described in other publications throughout the years; hence, it will not be the focus of this work (Sławiński et al., 2020; Sase et al., 2021; Cozma et al., 2022; Ganesh et al., 2022; Luo et al., 2023; Nardin et al., 2025; Tarantini et al., 2025; Sethi et al., 2026).

This narrative review aims to provide a comprehensive overview of CV toxicities associated with targeted and molecular therapies in NSCLC, focusing on shared pathophysiological mechanisms, drug class-specific clinical profiles, strategies for early diagnosis and risk stratification, and current approaches to prevention and management. By integrating translational and clinical evidence, this review seeks to support clinicians in optimizing CV safety while maintaining the efficacy of modern targeted therapies in NSCLC.

## 2 Review methodology

This narrative review was conducted to summarize and critically discuss current evidence on CV toxicities associated with targeted and molecular therapies in NSCLC, with a specific focus on underlying mechanisms, clinical manifestations, strategies for early diagnosis, prevention, and management. Given the heterogeneity of available data and the rapidly evolving therapeutic landscape, a narrative approach was chosen to integrate preclinical, translational, and clinical evidence across different drug classes.

A structured literature search was conducted using PubMed/MEDLINE and Embase databases, including publications up to 2025. Search terms included combinations of “non-small cell lung cancer”, “targeted therapy”, “tyrosine kinase inhibitors”, “antibody-drug conjugates”, “bispecific antibodies”, “cardiotoxicity”, “cardiovascular toxicity”, “QT prolongation”, “heart failure”, “hypertension”, and “cardio-oncology”. Original research articles, real-world studies, clinical trials, consensus statements, and relevant guidelines were considered. Review articles were used selectively to contextualize mechanistic concepts.

The review focused on targeted agents currently approved or under advanced clinical development for NSCLC, including TKIs, ADCs, and BsAbs therapies. Studies primarily addressing chemotherapy- or immune checkpoint inhibitor-related cardiotoxicity without relevance to targeted therapies were excluded due to the existence of comprehensive literature and due to the scope of our review. Evidence was synthesized qualitatively to highlight shared pathophysiological mechanisms, class-specific risk profiles, and clinical management strategies.

## 3 Common mechanisms of cardiovascular toxicity in targeted therapies for NSCLC

### 3.1 Endothelial dysfunction and vascular injury

**FIGURE 1:**
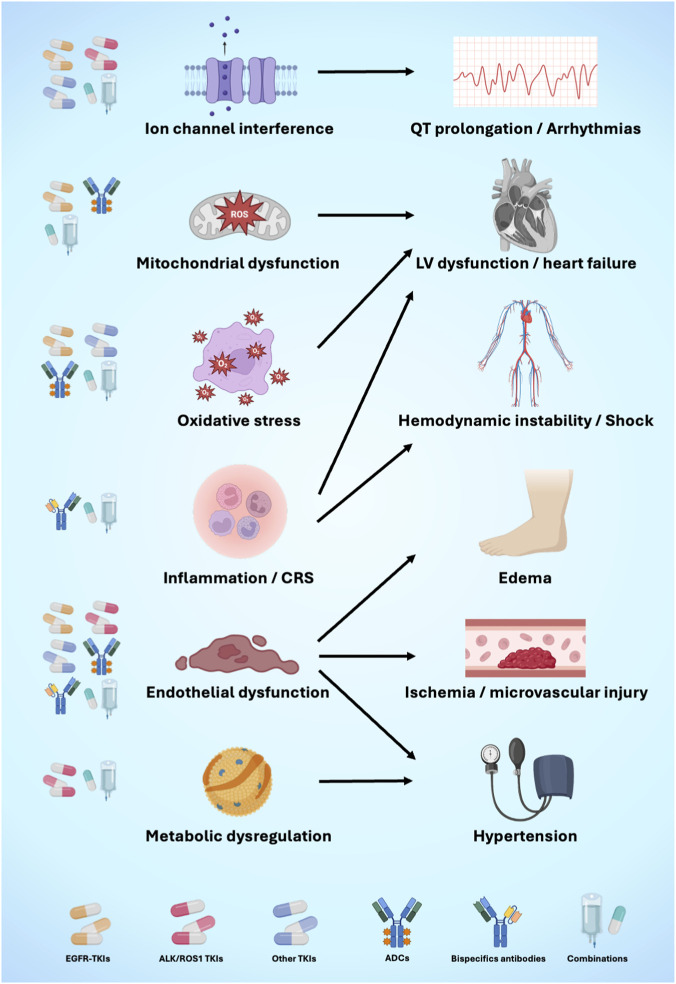
Shared biological mechanisms underlying cardiovascular toxicity of targeted therapies in NSCLC. Various therapeutic classes converge on common pathogenic pathways, including ion channel interference, mitochondrial dysfunction, oxidative stress, inflammatory activation, endothelial dysfunction, and metabolic dysregulation. These mechanisms contribute to overlapping cardiovascular phenotypes such as QT interval prolongation and arrhythmias, left ventricular dysfunction and heart failure, hemodynamic instability or shock, edema, ischemia and microvascular injury, and arterial hypertension. This mechanistic convergence supports a pathway-driven rather than drug-specific approach to cardiovascular risk assessment in NSCLC. ADC, antibody-drug conjugates; ALK, anaplastic lymphoma kinase; CRS, cytokine release syndrome; EGFR, epidermal growth factor receptor; LV, left ventricular. Created with Biorender.com.

Endothelial function refers to the vascular endothelium’s role in maintaining homeostasis by regulating vasomotion, inhibiting platelet aggregation and thrombus formation, and preserving barrier integrity, primarily through the release of endothelium-derived relaxing factors such as prostacyclin, nitric oxide (NO), and endothelium-derived hyperpolarizing factor (Quyyumi, 2006; Shimokawa, 2014). Endothelial activation can be triggered by several stimuli, including antineoplastic drugs (Hsu et al., 2021). The response starts with an inflammatory reaction that increases vascular permeability, thereby impairing vasomotor tone and leading to subsequent endothelial dysfunction (Figure 1). These events lead to a prothrombotic state and oxidative stress, which appears to play a key role in the shift from NO-mediated suppression of cellular activity to activation driven by redox signaling (Deanfield et al., 2007; Naderi-Meshkin and Setyaningsih, 2024). All of this leads to a state of change toward transformation, characterized by cellular senescence and programmed cell death, resulting in microvascular rarefaction (Naderi-Meshkin and Setyaningsih, 2024).

The consequences include the proliferation of vascular smooth muscle cells and neoangiogenesis with the formation of fragile vessels. This cascade promotes vascular remodeling, ultimately leading to organ dysfunction. It should also be noted that reduced NO bioavailability, together with increased production of reactive oxygen species (ROS) and peroxynitrite and elevated levels of thromboxane A_2_, contributes to the progression of atherosclerotic plaques and enhances platelet aggregation and thrombosis (Gallo and Savoia, 2024).

### 3.2 Oxidative stress, mitochondrial dysfunction, cardiomyocyte apoptosis

Oxidative stress occurs when the production of ROS exceeds the capacity of antioxidant detoxification systems. Excessive ROS can impair cell regeneration and induce lipid peroxidation, protein degradation, DNA damage, mitochondrial dysfunction, and disruptions in energy metabolism (Fei et al., 2022).

Mitochondria are both a major source and a primary target of ROS. Mitochondrial DNA is particularly vulnerable because it is located close to ROS production sites, lacks histone protection, and has limited repair capacity (LIM et al., 2005).

Mitochondrial ROS are generated by several proteins, including p66shc, monoamine oxidase (MAO)-A and MAO-B, and NADPH oxidase 4 (NOX4). P66shc, partially located in the mitochondrial intermembrane space, promotes ROS by oxidizing cytochrome C. MAO-A and MAO-B, located on the outer mitochondrial membrane, generate hydrogen peroxide during monoamine degradation. NOX4, situated within the inner mitochondrial membrane, produces superoxide and hydrogen peroxide, and its activity is regulated by ATP (Peoples et al., 2019).

Cardiomyocytes are heavily dependent on mitochondrial oxidative phosphorylation to meet the high energy demands of contraction and ion regulation; therefore, they are particularly vulnerable due to relatively weak endogenous antioxidant defenses (Bikomeye et al., 2022).

Excessive mitochondrial ROS can induce opening of the mitochondrial permeability transition pore, promote cell death, trigger mitochondrial DNA release and inflammatory signaling, and disrupt intracellular Ca^2+^ homeostasis (Fei et al., 2022). At the molecular level, interactions between mitochondrial membrane components, such as cardiolipin, ATP synthase, translocase of the outer membrane complex 20, and voltage-dependent anion channel, and the pathological protein aggregates impair complex I activity and reduce ATP production. This energy deficit promotes premature opening of the mitochondrial permeability transition pore, increasing cardiomyocyte death and contributing to cardiac dysfunction (Zong et al., 2024).

### 3.3 Impaired cardiac remodeling

The myocardial interstitium is a specialized cardiac tissue compartment that contains stromal cells supported by a highly complex extracellular matrix. This matrix comprises structural proteins, such as collagens and elastin, as well as nonstructural components, including glycoproteins, proteoglycans, and glycosaminoglycans (Díez et al., 2020). Cardiac fibroblasts regulate extracellular matrix deposition and maintain ventricular structural integrity, while influencing cardiomyocyte proliferation through fibronectin/β1-integrin signaling (Humeres and Frangogiannis, 2019).

Chronic cardiac stress compromises cardiomyocyte survival and metabolic signaling, including the phosphoinositide 3-kinase (PI3K)-Akt and the AMP-activated protein kinase (AMPK)–mTOR pathways, thereby promoting the release of pro-fibrotic mediators such as TGF-β, angiotensin II, and connective tissue growth factor, which drive persistent fibroblast activation and extracellular matrix deposition (Frangogiannis, 2019).

In response to diverse pathological stresses, cardiac fibroblasts exhibit marked phenotypic plasticity, transitioning from resident fibroblasts to activated states, including myofibroblasts characterized by induction of Acta2, Tagln, and extracellular matrix genes, and, at later stages after myocardial infarction, fibrocytes expressing matrix-stabilizing and osteochondral-associated genes such as Chad (Komuro et al., 2025).

Myocardial interstitial fibrosis plays a crucial role in heart failure (HF) by altering the myocardial structure, mechanics, and electrophysiological properties. Its impact on left ventricular ejection fraction (LVEF) depends not only on collagen accumulation but also on collagen composition, cross-linking, and spatial organization, contributing to increased myocardial stiffness (González et al., 2018). In addition, fibrosis creates an arrhythmogenic substrate and compromises myocardial oxygen delivery, further exacerbating cardiac dysfunction (Ravassa et al., 2023).

### 3.4 Effects on the cardiac conduction system and ion channels

The QT interval reflects the total duration of ventricular depolarization and repolarization. The duration of the cardiac action potential depends on the balance of multiple ion channels; reduced repolarizing K^+^ currents or increased depolarizing Na^+^ or Ca^2+^ currents can prolong the QT interval, providing the pathophysiological basis for prolongation of the QT interval (Schwartz et al., 2012).

The congenital condition, named long QT syndrome, is related to genetic variants of potassium or sodium channels, while the acquired disorder is most often a result of drug exposure (Gotta and Donner, 2025; Mondéjar-Parreño et al., 2025). QT prolongation represents a major risk factor for torsade de pointes and sudden cardiac death (Locati, 2006).

The gene KCNQ1 encodes the potassium v7.1 channel, which mediates the slow delayed rectifier potassium current (I_Ks_) and, together with the rapid delayed rectifier potassium current (I_Kr_), conducted by the potassium Kv11.1 channel encoded by KCNH2, named also hERG1, regulates the duration of the cardiac action potential repolarization phase. Pharmacological inhibition of the pore-forming α-subunit of the I_Kr_/hERG channel by commonly used drugs can impair this process, leading to drug-induced long QT syndrome (El Harchi and Brincourt, 2022).

Beyond the mechanisms directly responsible for QT prolongation, targeted therapies may exert additional effects on cardiac electrophysiology, including sinus node dysfunction, atrioventricular conduction disturbances, atrial and ventricular arrhythmias, and autonomic imbalance. These findings reflect a broader impact on the cardiac conduction system and overall electrical stability, rather than being exclusively attributable to QT interval prolongation (Keramida et al., 2025).

### 3.5 Effects on vascular tone and blood pressure

Vascular tone, defined as the contractile state of vascular smooth muscle cells in small arteries and arterioles, is a primary determinant of vascular resistance. As such, it plays a central role in regulating blood pressure (BP) and distributing blood flow across tissues and organs (Jackson, 2000).

Vascular endothelial growth factors (VEGFs) are secreted proteins essential for vascular growth and homeostasis, acting through endothelial VEGF receptors (Camarda et al., 2022).

VEGF regulates vascular tone primarily by inducing vasodilatory mediators. Activation of VEGF receptor 2 stimulates PI3K/Akt signaling, leading to enhanced endothelial NO synthase activity and NO production, which promotes endothelial survival, vascular permeability, and endothelium-dependent vasodilation. VEGF signaling also induces prostacyclin production, further contributing to vasodilation (Pandey et al., 2018).

VEGF-related hypertension is primarily attributed to reduced NO production in arterioles and other resistance vessels, due to downregulation of endothelial NO synthase following VEGF inhibition. The resulting decrease in NO, an important vasodilator, leads to vasoconstriction, increased peripheral resistance, and elevated BP (Kamba and McDonald, 2007). Also, the VEGF-related suppression of nephrin, a transmembrane protein essential for maintaining the integrity of the glomerular slit diaphragm, may potentially contribute to hypertension and proteinuria (Curigliano et al., 2016).

### 3.6 Role of inflammation and cytokine release syndrome

BsAbs combine the specificities of two antibodies, enabling simultaneous binding to distinct antigens. Bispecific T-cell engagers (BiTEs) are a subclass of BsAbs composed of two linked single-chain variable fragments with 1 + 1 binding valency. These small, flexible molecules physically bridge T cells and tumor cells, thereby inducing T-cell cytotoxicity and cytokine release only when both binding sites are engaged, and effectively directing T-cell–mediated tumor cell killing (Smits and Sentman, 2016).

Cytokine release syndrome (CRS) arises from this potent immune activation and is triggered by the engagement of BiTEs, chimeric antigen receptor T (CAR-T) cells, or immune checkpoint inhibitors with their target antigens. This process activates both target and bystander immune and nonimmune cells, leading to the release of multiple proinflammatory cytokines, including IL-6, IFN-γ, and TNF-α, primarily derived from B and T lymphocytes and NK cells. CRS is further amplified by interactions with endothelial cells, monocytes/macrophages, and dendritic cells, which intensify cytokine release and contribute to increased clinical severity and organ damage (Synnott et al., 2025).

From a CV perspective, the systemic inflammatory response characteristic of CRS has profound hemodynamic and myocardial consequences. Elevated cytokine levels induce widespread endothelial activation and increased vascular permeability, leading to intravascular volume depletion, hypotension, and impaired tissue perfusion (Lee et al., 2019). Concomitantly, NO overproduction and dysregulated vasodilatory signaling further contribute to systemic vasodilation and hemodynamic instability (Neelapu et al., 2018).

At the myocardial level, proinflammatory cytokines exert direct negative inotropic effects, impair calcium handling, and promote mitochondrial dysfunction, thereby reducing cardiac contractile reserve (Norelli et al., 2018). These mechanisms may precipitate acute HF, arrhythmias, and, in severe cases, cardiogenic shock, particularly in patients with pre-existing CV disease or limited cardiac reserve (Alvi et al., 2019). Moreover, sustained inflammatory signaling may promote myocardial edema and transient or persistent ventricular dysfunction, mimicking stress-induced cardiomyopathy (Ganatra et al., 2019).

## 4 Class-specific cardiovascular toxicities

The increasing use of molecular targeted therapies, ADCs, and combination strategies has significantly improved clinical outcomes in patients with NSCLC. However, these therapeutic advances have also led to a growing incidence of CV toxicities, which may limit treatment adherence, compromise quality of life, and negatively affect overall prognosis. Unlike conventional cytotoxic chemotherapy, CV adverse events related to targeted agents often arise from specific biological interactions with signaling pathways involved in cardiac homeostasis, vascular integrity, and electrophysiological stability (Table 1).

**TABLE 1:** Targeted therapies in NSCLC: mechanism of action and main cardiovascular toxicities.

Drug class	Drug agent	Mechanism of action	Main cardiovascular toxicities
EGFR-TKIs	Erlotinib, gefitinib, afatinib, osimertinib	Inhibition of EGFR signaling	LV dysfunction, heart failure (mainly with osimertinib); QT prolongation (uncommon)
ALK/ROS1-TKIs	Crizotinib, alectinib, brigatinib, lorlatinib	Inhibition of ALK/ROS1 fusion proteins	Bradycardia, conduction disorders, QT prolongation; hypertension; dyslipidemia (lorlatinib)
RET inhibitors	Selpercatinib, pralsetinib	Selective RET inhibition	Hypertension, QT prolongation; vascular events (rare)
MET inhibitors	Capmatinib, tepotinib	MET pathway inhibition	Peripheral edema
NTRK inhibitors	Larotrectinib, entrectinib	Inhibition of TRK fusion proteins	QT prolongation (rare)
BRAF/MEK inhibitors	Dabrafenib + trametinib	MAPK pathway inhibition	LV dysfunction, hypertension, arrhythmias
KRAS G12C inhibitors	Sotorasib, adagrasib	Irreversible KRAS G12C inhibition	QT prolongation (rare)
HER2-targeted ADCs	Trastuzumab deruxtecan	HER2 targeting with cytotoxic payload	LV dysfunction
Bispecific antibodies/BiTEs	Ivonescimab, amivantamab	Dual antigen targeting with immune activation	CRS, hypotension, arrhythmias, hemodynamic instability, thrombosis
Combination strategies	TKI + ICI/chemotherapy	Multi-pathway inhibition	Myocarditis (rare), hypertension, LV dysfunction, increased vascular risk

ADC, antibody-drug conjugates; ALK, anaplastic lymphoma kinase; BiTE, bispecific T-cell engager; CRS, cytokine release syndrome; EGFR, epidermal growth factor receptor; HER2, human epidermal growth factor 2; ICI, immune-checkpoint inhibitor; LV, left ventricular; MAPK , mitogen-activated protein kinase; NTRK, neurotrophic tyrosine receptor kinase; TKIs, tyrosine kinase inhibitors.

### 4.1 EGFR-tyrosine kinase inhibitors

Epidermal growth factor receptor (EGFR) TKIs, including erlotinib, gefitinib, afatinib, and osimertinib, represent the standard of care for patients with EGFR-mutant NSCLC (Hendriks et al., 2023; Hendriks et al., 2025). These agents exert their antitumor activity by inhibiting aberrant EGFR signaling, thereby suppressing tumor cell proliferation and survival and often enabling sustained disease control with chronic treatment exposure. As a consequence, even low-grade CV toxicities may become clinically significant over time.

**FIGURE 2:**
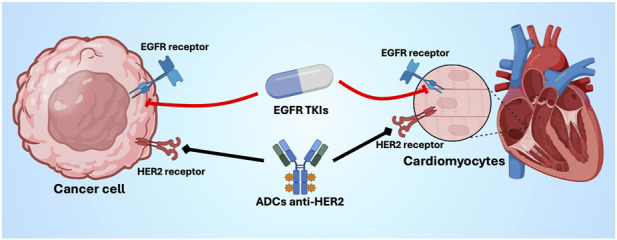
EGFR/ErbB signaling in cancer cells and cardiomyocytes. Overlapping targeting of EGFR and HER2 signaling pathways in cancer cells and cardiomyocytes. EGFR tyrosine kinase inhibitors suppress oncogenic activity. EGFR signaling in tumor cells, but may also inhibit cardiomyocyte EGFR-mediated survival pathways. ADC, antibody-drug conjugates; EGFR, epidermal growth factor receptor; HER2, human epidermal growth factor 2; TKI, tyrosine kinase inhibitors. Created with Biorender.com.

Beyond tumor biology, EGFR and related ErbB receptors are expressed in cardiomyocytes, playing a critical role in myocardial survival signaling and adaptive responses to mechanical and oxidative stress (Zhu et al., 2010) (Figure 2). Inhibition of the EGFR/ErbB network may therefore impair cardioprotective pathways, increasing myocardial susceptibility to injury and functional deterioration, particularly in patients with limited cardiac reserve or pre-existing CV disease (Wang et al., 2024b). Prior thoracic radiotherapy may further amplify this vulnerability (Wang et al., 2024a).

Among EGFR-TKIs, osimertinib has been most consistently associated with clinically meaningful cardiotoxicity, including reductions in LVEF and isolated cases of overt HF (Kunimasa et al., 2021). Although earlier-generation EGFR-TKIs have been sporadically linked to CV adverse events, the signal appears more pronounced with osimertinib, possibly reflecting both off-target effects and longer treatment duration (Wang et al., 2024b). The underlying mechanisms are multifactorial, involving the disruption of ErbB-mediated cardiomyocyte survival, mitochondrial dysfunction, and increased susceptibility to oxidative stress. In parallel, EGFR-TKIs may interfere with cardiac ion channels, particularly the hERG potassium channel, and L-type Ca^2+^ channels, leading to prolongation of the QT interval and an increased risk of ventricular arrhythmias (Crone et al., 2002; Sayegh et al., 2023).

In addition to direct myocardial effects, EGFR-TKIs have been associated with endothelial dysfunction and impaired NO-mediated vasodilation (Moraes et al., 2014). While most CV adverse events reported with EGFR-TKIs are mild to moderate, severe toxicity requiring dose modification or treatment interruption has been described in real-world and post-marketing settings, particularly in patients receiving long-term therapy (Sayegh et al., 2023).

### 4.2 ALK/ROS1 tyrosine kinase inhibitors

Anaplastic lymphoma kinase (ALK) and ROS1 inhibitors, such as crizotinib, alectinib, brigatinib, and lorlatinib, have dramatically improved outcomes in patients with ALK- or ROS1-rearranged NSCLC (Peng et al., 2023). These agents are often administered for prolonged periods, resulting in prolonged exposure that may unveil treatment-related CV effects even when initially mild. They selectively inhibit oncogenic fusion proteins that drive tumor growth and survival.

**FIGURE 3:**
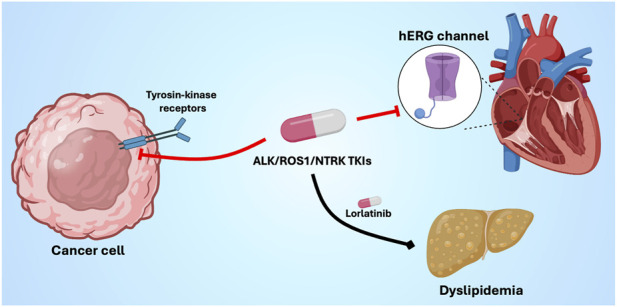
Antitumor activity, cardiovascular, and metabolic effects of ALK/ROS1/NTRK TKIs. On-target antitumor and off-target cardiovascular and metabolic effects of ALK/ROS1/NTRK tyrosine kinase inhibitors. These agents inhibit oncogenic tyrosine kinase receptors in cancer cells, whereas off-target interactions in cardiomyocytes may impair cardiac repolarization by inhibiting the hERG potassium channel, leading to QT interval prolongation and arrhythmias. In parallel, lorlatinib exerts hepatic metabolic effects that disrupt lipid homeostasis, resulting in clinically relevant dyslipidemia. ALK, anaplastic lymphoma kinase; NTRK, neurotrophic tyrosine receptor kinase; TKI, tyrosine kinase inhibitors. Created with Biorender.com.

A characteristic feature of ALK/ROS1-TKIs is their impact on cardiac rhythm and conduction. Bradycardia and atrioventricular conduction disturbances are frequently observed, particularly with crizotinib and alectinib, and are thought to result from off-target modulation of autonomic regulation and cardiac ion channel activity (Cirne et al., 2021) (Figure 3).

QT interval prolongation, although less common, has been reported, particularly with lorlatinib, reflecting additional effects on cardiac repolarization pathways (Nagasaka et al., 2020). While the absolute risk of malignant ventricular arrhythmias appears low, electrolyte abnormalities or impaired hepatic metabolism may increase it. Lorlatinib is also associated with pronounced dyslipidemia, including hypercholesterolemia and hypertriglyceridemia, enhancing patients’ CV risk profile (Arriola et al., 2024). Brigatinib has been linked to arterial hypertension and, in rare cases, thromboembolic events, potentially related to endothelial dysfunction and vascular remodeling (Cai et al., 2025). These vascular effects appear less frequent than rhythm disturbances but may assume greater relevance in patients with pre-existing CV risk factors or prior vascular disease. Overall, CV toxicities associated with ALK and ROS1 inhibitors are usually manageable but require proactive electrocardiographic and metabolic monitoring, as well as individualized CV risk assessment to ensure treatment continuity without compromising patient safety.

### 4.3 RET, MET, NTRK, BRAF, KRAS-G12C inhibitors

The expanding landscape of actionable molecular alterations in NSCLC has led to the introduction of several additional targeted agents with heterogeneous CV safety profiles. Although these therapies are often perceived as having a more favorable CV risk compared with earlier-generation TKIs, their increasing use and prolonged administration have revealed distinct class-specific toxicities that warrant careful clinical attention.

Selective RET inhibitors, such as selpercatinib and pralsetinib, are associated with arterial hypertension and QT interval prolongation (Nardo et al., 2023; Nguyen and Geirnaert, 2023). Hypertension is the most frequent CV adverse event and may emerge early during treatment, sharing those mechanisms observed with VEGF TKIs (Hou et al., 2025). QT prolongation is thought to arise from interference with cardiac repolarization channels (Nardo et al., 2023). Moreover, selpercatinib is associated with an increased risk of thrombotic events (Hou et al., 2025) mesenchymal-epidermal transition (MET) inhibitors, including capmatinib and tepotinib, are primarily associated with peripheral edema due to altered capillary permeability and lymphatic drainage rather than direct myocardial injury (Nishio et al., 2022). This fluid retention phenotype may mimic or exacerbate HF symptoms, especially in patients with underlying left ventricular (LV) dysfunction. While often manageable with supportive measures, edema may precipitate clinical decompensation, underscoring the importance of differentiating drug-related volume redistribution from true cardiac dysfunction through careful clinical, laboratory, and echocardiographic assessment.

Neurotrophic tyrosine receptor kinase (NTRK) inhibitors, such as larotrectinib and entrectinib, are generally well tolerated from a CV perspective. Nevertheless, entrectinib has been associated with QT prolongation and rare cases of HF, potentially related to mitochondrial stress and ion channel modulation (Dunn and PharmD, 2020). Although these events are uncommon, their occurrence underscores the need for vigilance during long-term therapy, particularly in patients with additional CV risk factors.

Similarly, BRAF inhibitors used in combination with MEK inhibitors for BRAF V600E-mutant NSCLC may impair cardiac function by inhibiting the mitogen-activated protein kinase/extracellular signal-regulated kinase (MAPK/ERK) pathway, which plays a central role in cardiomyocyte adaptation to stress. This interference can result in reduced LVEF, arterial hypertension, and, less frequently, arrhythmias, underscoring the importance of this signaling axis in myocardial homeostasis (Glen et al., 2022).

KRAS G12C inhibitors, including sotorasib and adagrasib, constitute a relatively new class of targeted therapies with evolving CV safety data (El Zaitouni et al., 2025). QT prolongation and arrhythmias have been reported, often in association with electrolyte disturbances or clinically relevant drug–drug interactions, particularly with agents affecting CYP-mediated metabolism (Malhotra et al., 2024). Given the limited long-term CV data available, careful baseline assessment and early on-treatment monitoring are recommended, especially during the early phases of treatment.

### 4.4 Antibody-drug conjugates

ADCs have emerged as an innovative therapeutic approach in NSCLC, combining the target specificity of monoclonal antibodies with the potent cytotoxic activity of chemotherapy payloads, thereby expanding treatment options for molecularly defined subgroups. Trastuzumab deruxtecan (T-DXd) is approved for human epidermal growth factor receptor 2 (HER2)-mutant NSCLC and combines a monoclonal antibody targeting HER2 with a cytotoxic topoisomerase I inhibitor payload (Jänne et al., 2025). HER2 is not only a tumor-associated receptor but also a key component of cardioprotective signaling pathways through the neuregulin-ErbB axis. In cardiomyocytes, HER2 signaling supports cell survival, mitochondrial integrity, and adaptive responses to mechanical stress (Roskoski, 2014). Inhibition of HER2 may therefore compromise myocardial cell survival, leading to reductions in LVEF and overt HF, through mechanisms analogous to those observed with trastuzumab in breast cancer. Although the incidence of overt cardiotoxicity with T-DXd in NSCLC appears relatively low, reported cases highlight the need for careful CV surveillance, particularly in patients with prior exposure to cardiotoxic therapies or baseline cardiac disease (Smit et al., 2024).

In addition to target-related effects, the cytotoxic payload of ADCs may contribute to CV toxicity through indirect mechanisms, including mitochondrial stress, oxidative damage, and endothelial dysfunction (Long et al., 2024). These effects may be amplified by the high drug–antibody ratio and bystander effect characteristic of next-generation ADCs, potentially increasing off-target exposure of cardiac and vascular tissues.

For ADCs targeting TROP2, such as datopotamab deruxtecan, cardiotoxicity appears less target-dependent and more related to indirect mechanisms, including systemic inflammation, oxidative stress, and treatment combinations (Nguyen et al., 2023). While current clinical data suggest a favorable CV safety profile, experience with these agents in NSCLC remains limited, and long-term CV outcomes have yet to be fully elucidated.

Taken together, ADC-associated CV toxicity reflects the complex interplay between target biology, payload characteristics, and patient-specific risk factors (Ciappina et al., 2025). As clinical experience expands and ADCs are increasingly used in earlier treatment lines and in combination regimens, continued vigilance and structured CV monitoring will be essential to identify emerging safety signals and optimize long-term outcomes promptly.

### 4.5 Bispecific antibodies

BsAbs are therapies designed to simultaneously bind two distinct antigens or epitopes, thereby enabling coordinated modulation of multiple biological pathways or cellular interactions within the tumor microenvironment (Klein et al., 2024).

Ivonescimab is a novel BsAbs targeting PD-1 and VEGF that is currently being evaluated in different clinical settings for NSCLC, both as monotherapy and in combination with chemotherapy (Fang et al., 2024; Chen et al., 2025; Xiong et al., 2025). The main cardiovascular adverse events appear to be primarily driven by the anti-VEGF component and include arterial hypertension, arterial thromboembolic events, bleeding complications such as gingival or ophthalmic hemorrhage, and, less frequently, congestive heart failure.

Amivantamab is a BsAbs that binds both EGFR and MET. It has currently been evaluated in patients with EGFR-mutated tumors, both alone and in combination with lazertinib or chemotherapy (Zhou et al., 2023; Cho et al., 2024; Passaro et al., 2024; Yang et al., 2025). Principal CV toxicities are linked to the anti-MET compound, with a higher risk of pulmonary embolism, deep vein thrombosis, and cardiac failure. Moreover, particularly during the first two cycles, it is associated with infusion-related reactions that can cause fever, tachycardia, and hypotension (Papassotiriou et al., 2025).

BiTEs are emerging BsAbs in NSCLC characterized by immune-mediated mechanisms of action. Unlike conventional targeted therapies, these agents exert their antitumor activity by redirecting endogenous T cells toward tumor cells through simultaneous engagement of both immune and tumor-associated antigens, thereby inducing rapid and potent immune activation. By simultaneously engaging T cells and tumor antigens, these agents can induce CRS. Furthermore, endothelial activation and NO overproduction further contribute to circulatory instability and impaired tissue perfusion (Arvanitis et al., 2025). In severe cases, CRS may involve the CV system more extensively, resulting in myocardial depression, arrhythmias, and cardiogenic shock (Shalabi et al., 2020). These manifestations reflect the combined effects of inflammatory cytokines on myocardial contractility, calcium handling, and mitochondrial function, as well as the hemodynamic consequences of systemic vasoplegia. In some patients, transient or persistent ventricular dysfunction has been reported, occasionally resembling stress-induced cardiomyopathy, with variable reversibility after resolution of the inflammatory process (Ganatra et al., 2022).

Although CV complications associated with BsAbs are relatively infrequent, they tend to occur early during treatment. They may evolve rapidly, particularly in patients with limited cardiac reserve or pre-existing CV disease.

### 4.6 Combination therapies

Combining strategies, including targeted therapies with immunotherapy or chemotherapy, poses an additional challenge from a CV perspective. These approaches are increasingly adopted to enhance the depth and durability of tumor response, particularly in advanced or refractory disease settings, but inevitably increase the complexity of CV risk assessment. Synergistic antitumor activity may be accompanied by additive or potentiated CV toxicity, reflecting the convergence of distinct but overlapping pathogenic mechanisms.

The combination of TKIs with immune checkpoint inhibitors may enhance systemic inflammation, increasing the risk of myocarditis, arrhythmias, and HF (Ciccarese et al., 2024).

TKI-chemotherapy combinations may exacerbate oxidative stress and mitochondrial dysfunction, leading to cumulative myocardial injury. This effect may be particularly relevant when TKIs are combined with platinum-based regimens or anthracycline-containing protocols, in which subclinical myocardial damage may accumulate over time and manifest as delayed, reduced LVEF (Tan et al., 2025). The overlapping impact of these agents on mitochondrial integrity and redox balance underscores the importance of longitudinal cardiac surveillance.

In selected settings, targeted agents combined with anti-angiogenic therapies, such as bevacizumab, may further amplify endothelial dysfunction, resulting in arterial hypertension and thromboembolic complications (Totzeck et al., 2017). To note, treatment with docetaxel plus ramucirumab, another anti-VEGF, is burdened with 30% risk of bleeding or hemorrhage, in addition to hypertension, congestive HF, and both venous and arterial thromboembolism (Garon et al., 2014).

Nintedanib, an oral multi-TKI agent that targets platelet-derived growth factor receptor (PDGFR)-α, PDGFR-β, fibroblast growth factor receptor (FGFR) 1-3, and VEGF receptors 1-3, is also used in combination with docetaxel (Reck et al., 2014). The VEGF inhibition leads to ad 14% risk of bleeding, 3.5% hypertension, while thromboembolic events are up to 5%.

The coexistence of VEGF pathway inhibition and targeted oncogenic signaling blockade may also impair vascular repair mechanisms, thereby increasing susceptibility to ischemic events, particularly in patients at high risk before therapy (Mihalcea et al., 2023).

## 5 Primary prevention of cardiovascular toxicities

Prevention strategies for CV disease constitute a cornerstone in the management of cancer patients. Protective measures may be implemented as primary or secondary prevention, depending on the timing of assessment and the patients’ medical histories. Primary prevention refers to approaches aimed at avoiding or minimizing the risk of CV damage from cancer therapy in patients without known CV disease. On the other hand, secondary prevention encompasses the overall strategy for patients with pre-existing CV disease, prior cancer treatment-related cardiovascular disease (CTRCD), and new onset evolving CV disease during cancer therapy.

### 5.1 General considerations

Minimizing exposure to cardiotoxic agents whenever oncologically feasible: if an equally effective alternative therapy with a better cardiac safety profile is available, it should be chosen for high-risk patients. In the context of NSCLC-targeted therapies, an oncologist might select a less cardiotoxic TKI or, if possible, adjust the dose. For instance, because certain ALK inhibitors are associated with a higher incidence of bradyarrhythmia than others, if a patient has underlying conduction disease, a switch to an alternative ALK inhibitor or closer monitoring could prevent serious complications.

Importantly, prophylactic cardioprotective therapy may be warranted in select high-risk cases. Physicians should consider renin-angiotensin system inhibitors (RASi), such as angiotensin converting enzyme inhibitors (ACEi) or angiotensin receptor blockers (ARB), and beta-blockers for primary prevention in patients at high or very high CV risk who are receiving cancer therapies known to cause HF: evidence suggests that these medications may attenuate a cardiotoxic decline in LVEF. Although direct trial data in TKI recipients are lacking, this preventative approach is supported by analogy to other anti-tumoral drugs; thus, international guidelines extend this recommendation to targeted therapies with potential HF risk (Lyon et al., 2022).

Similarly, statins have emerged as potential cardioprotective agents. They can be introduced in cancer patients at high or very high CV risk as a primary prevention measure, even in the absence of elevated low-density lipoprotein cholesterol levels (Calvillo-Argüelles et al., 2019). This is particularly relevant for NSCLC patients receiving VEGF inhibitors or TKIs that promote atherosclerotic processes (Yeh and Bickford, 2009): statin pleiotropic effects help to stabilize plaques and improve endothelial function (Almeida and Budoff, 2019; Zhang et al., 2024).

### 5.2 Traditional cardiovascular risk factors

Patients without prior CV disease or CTRCD benefit from common general strategies of CV prevention (Visseren et al., 2021). Main causal and modifiable risk factors involved in atherosclerotic disease are low-density lipoprotein cholesterol, high BP, cigarette smoking, and diabetes mellitus.

The direct causal role of low-density lipoprotein cholesterol is well established, and its blood levels, as well as the duration of elevated levels, are directly associated with the progression and severity of atherosclerosis. The threshold for reduction actions, pharmacological and not, should be targeted according to the overall risk profile of patients (Mach et al., 2025a). Cigarette smoking accounts for about half of avoidable deaths in smokers, half of whom involve atherosclerosis (Doll et al., 2004). Beyond the mortality, the overall burden of morbidity related to cigarette smoking remains considerable (GBD, 2017 Risk Factor Collaborators, 2018). Higher BP values constitute a major risk factor for coronary artery disease, HF, chronic kidney disease, and atrial fibrillation (McEvoy et al., 2024). With specific regard to NSCLC, data from observational studies report a significant role of diabetes mellitus and history of smoking in predicting cancer-related treatment CV toxicity among patients receiving different schemas of target therapy (Kunimasa et al., 2020; Kobat et al., 2024).

### 5.3 Cardiovascular risk assessment

Cardiologist guidelines have updated the focus of CV prevention, shifting from the sole CV death to overall morbidity, including CV fatal and non-fatal events: myocardial infarction and stroke. To define the individual CV risk, the SCORE2 and SCORE2-OP, for people aged 40–69 years old and ≥70 years old, respectively, represent the standard tools (Visseren et al., 2021).

The Heart Failure Association and the International Cardio-Oncology Society have developed specific tools to stratify the risk of should be considered to stratify CV toxicity risk in patients with cancer scheduled to receive specific cardiotoxic anticancer therapy (Lyon et al., 2020; Gynnild et al., 2024): patients are categorized as low, moderate, high or very high risk for cardiotoxicity based on age, comorbidities, and the specific cancer therapy planned, including anthracyclines, HER2-targeted therapies, VEGF inhibitors, or RAF and MEK inhibitors.

Any baseline ECG abnormalities warrant referral to a cardiologist before any therapy begins. Transthoracic echocardiography is advised for patients stratified as high or very high CV risk before starting cancer treatment (Lyon et al., 2022). Baseline measurement of cardiac biomarkers, troponin, and natriuretic peptides, is recommended in patients at risk, if those markers will be used for monitoring during treatment.

## 6 Secondary prevention

Secondary prevention refers to efforts to prevent the progression or recurrence of CV disease in patients with established or pre-existing conditions. In patients with NSCLC receiving targeted therapies, secondary prevention encompasses the management of therapy-induced cardiotoxicity and the care of underlying cardiac conditions throughout cancer treatment. Optimal treatment of conditions like coronary artery disease, HF, or atrial fibrillation should continue uninterrupted, with necessary modifications to avoid drug interactions with oncology treatments (Patil et al., 2025).

Cardiological evaluation before starting cancer treatment for NSCLC is recommended for all patients with pre-existing CV disease. Main aims include optimizing cardiac medication and addressing unstable cardiac issues such as pending revascularization or arrhythmia control. This ensures the patient begins cancer therapy on a stable cardiac basis.

The follow-up during the targeted therapy is essential to early intercept possible CTRCD and promptly manage them. Specific adverse events will be discussed below, but the common principle is not to delay cardioprotective treatment. As soon as a cardiac issue is detected, appropriate therapy should be initiated, along with any necessary modifications to oncological treatment. Early intervention can prevent progression to overt CV disease and may allow the patient to continue life-saving targeted therapy (Curigliano et al., 2020).

Secondary prevention extends into the post-therapy survivorship period. Patients who have experienced any CV toxicity during treatment or with prior CV disease should undergo a formal cardiac evaluation at the end of targeted therapy. Moreover, long-term cardiological follow-up is recommended for patients at high or very high risk of CV disease, even if they have not experienced an adverse event (Banke et al., 2018). Of note, tomost of this evidence is limited to non-targeted therapy and in cancer types with higher overall survival; therefore, data in NSCLC are limited, and recommendations for this setting are based more on analogy than on robust evidence. General recommendations suggest follow-up echocardiography and biomarker assessment at 3 months and 12 months after completion of cancer therapy (Lyon et al., 2022).

## 7 Management of main cardiovascular adverse events

### 7.1 QTc prolongation

**FIGURE 4:**
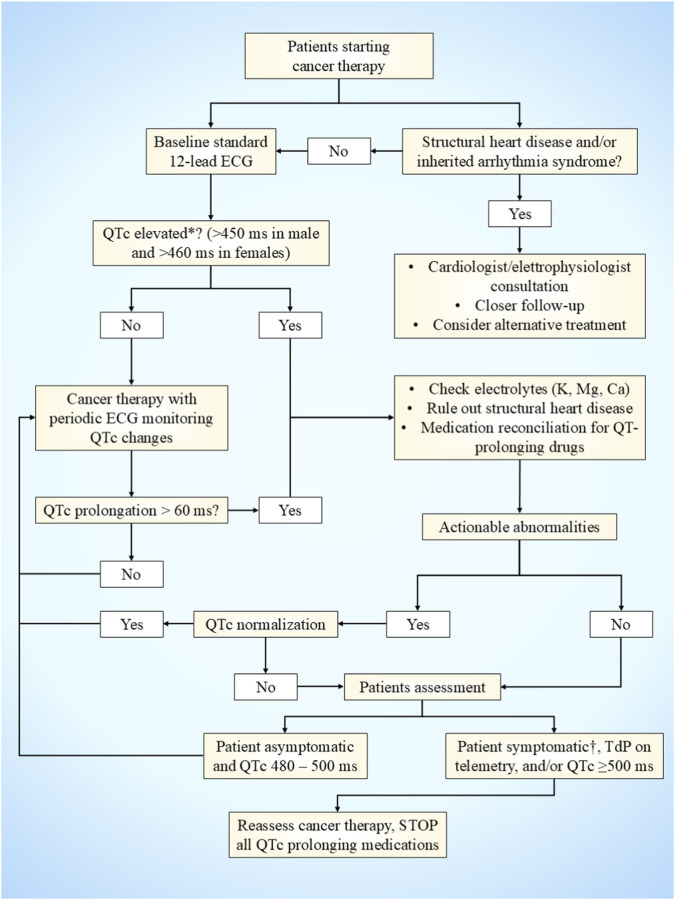
QTc prolongation management. The figure depicts a flowchart for the management of QTc prolongation in cancer patients receiving targeted therapy. *manually evaluated using Fridericia correction formula; ^†^dizziness, near syncope, hypotension, chest pain, or palpitations. TdP, torsade de pointes.

QT interval prolongation is a recognized CV adverse effect of several targeted therapies used in NSCLC. Although the incidence of malignant ventricular arrhythmias remains low, corrected QT (QTc) prolongation is clinically relevant due to its association with torsade de pointes (TdP) and sudden cardiac death. Effective management relies on structured risk assessment, standardized monitoring, prompt correction of reversible factors, and judicious modification of cancer therapy when required (Figure 4).

A baseline 12-lead ECG must be obtained before initiating any QT-prolonging anticancer therapy, including targeted agents, with QT correction preferably calculated using the Fridericia formula due to its superior accuracy at varying heart rates (Fradley and Moslehi, 2015). QTc prolongation is generally defined as >450 ms in men and >460 ms in women (Schwartz et al., 1993), with QTc ≥500 ms or an increase of ≥60 ms from baseline representing high-risk thresholds (Zeppenfeld et al., 2022). However, recent International Cardio-Oncology Society consensus defining CV toxicities of cancer therapies suggests against using the ≥60 ms increase cutoff when the QTc persists <500 ms, given the absence of evidence of increased arrhythmia incidence. Conversely, a QTc curation between 480 and 500 ms mandates closer electrocardiographic monitoring (Herrmann et al., 2022).

The baseline evaluation should also include identification of predisposing factors such as structural heart disease, congenital long QT syndrome, electrolyte abnormalities, renal or hepatic dysfunction, and concomitant use of non-oncologic QT-prolonging medications (Woosley et al., n.d.). Evidence from systematic reviews indicates that while QT prolongation with targeted therapies is relatively common, progression to life-threatening arrhythmias is rare when appropriate management strategies are applied (Porta-Sánchez et al., 2017). In patients with asymptomatic QTc prolongation below 500 ms, cancer therapy can usually be continued with intensified monitoring, provided that reversible contributors are promptly addressed (Kim et al., 2021; Herrmann et al., 2022). Management priorities include aggressive correction of electrolyte abnormalities, targeting serum potassium levels >4.0–4.5 mmol/L and magnesium >2.0 mg/dL, and discontinuation or substitution of non-essential QT-prolonging drugs. Hypocalcemia, hypothyroidism, and dehydration should also be addressed.

If QTc approaches 500 ms, reassessment of the oncologic regimen is warranted. Temporary dose interruption or reduction of the targeted agent should be considered, particularly for drugs with known dose-dependent QT effects such as multitarget TKIs (Sayegh et al., 2023). Importantly, inappropriate permanent discontinuation of effective cancer therapy should be avoided when QT prolongation is < 500 ms, reversible causes are identifiable, and patients remain asymptomatic. QTc ≥500 ms represents a high-risk condition requiring immediate intervention. If TdP or sustained ventricular arrhythmia occurs, management follows advanced cardiac life support principles, including intravenous magnesium administration regardless of baseline magnesium levels, correction of bradycardia, and electrical cardioversion if hemodynamic instability is present (Curigliano et al., 2020).

Rechallenge with the offending targeted therapy may be considered only after careful, multidisciplinary evaluation, with QTc normalization and elimination of modifiable risk factors. If treatment is resumed, it should be continued with enhanced ECG monitoring and, where possible, with dose reduction or with alternative agents with a lower impact on the QT interval (Curigliano et al., 2020).

### 7.2 Systemic arterial hypertension

Systemic Arterial hypertension is one of the most frequent CV toxicities observed in patients with NSCLC treated with targeted therapies, particularly TKIs interfering with the VEGF signaling pathway and, to a lesser extent, ALK and RET inhibitors. Although hypertension is relatively common in oncological populations, pre-existing hypertension, older age, and renal dysfunction represent the main predictors of clinically relevant treatment-emergent hypertension during targeted therapy.

The definition of hypertension and its therapeutic strategies are largely based on broader general hypertension guidelines and recommendations, which have not been systematically and sufficiently validated in cancer patients (McEvoy et al., 2024). Baseline BP assessment is mandatory before treatment initiation, and BP should be maintained below 140/90 mmHg, ideally <130/80 mmHg, throughout therapy. Notably, target BP values may be lowered before starting a VEGF inhibitor to minimize the additive hypertensive effect of these drugs (Caletti et al., 2018): bevacizumab per se is associated with an increased risk of hypertension ranging from 3 to 7.5 times, from low to high doses, respectively (Zhu et al., 2007). Higher BP values are accepted in patients with metastatic cancer and poor estimated prognosis (Lyon et al., 2022).

During treatment, regular BP monitoring is recommended, especially in the first weeks after initiation or dose escalation. In patients who develop new-onset or worsening hypertension, after having addressed potentially reversible elevated BP values contributors, pharmacological management follows general hypertension guidelines: ACEi or ARB are recommended as first-line agents, given their favorable CV profile and potential protective effects on cardiac dysfunction. Dihydropyridine calcium channel blockers may be used as second-line agents or in combination therapy, if BP remains ≥160/100 mmHg (McEvoy et al., 2024). Non-dihydropyridine calcium channel blockers should generally be avoided due to drug-drug interactions mediated by CYP3A4 inhibition and P-glycoprotein (Beavers et al., 2022). Beta-blockers, diuretics, or other antihypertensive agents may be considered based on comorbidities and clinical context.

In cases of severe hypertension, typically systolic BP ≥ 180 mmHg and/or diastolic BP ≥ 110 mmHg, targeted therapy associated with BP elevation should be temporarily withheld until adequate BP control is achieved, ≤160/100 mmHg. Once BP is controlled, cancer therapy may be safely resumed, often with dose reduction and intensified monitoring.

Upon completion of anticancer therapy, patients may require a down-titration of antihypertensive drugs to avoid rebound hypotension and, thus, ischemic events. Over the long term, the prevalence of hypertension in cancer survivors is higher than in the general population (Armstrong et al., 2013).

### 7.3 Dyslipidemia

Despite its relatively limited short-term clinical impact, dyslipidemia has emerged as a significant adverse effect of targeted therapies. While second-generation ALK inhibitors are associated with a relatively modest metabolic impact, the third-generation inhibitor lorlatinib is characterized by a high incidence of hypercholesterolemia and hypertriglyceridemia (Shaw et al., 2020) Lorlatinib-induced hypercholesterolemia and hypertriglyceridemia occur in more than 80% and 60% of patients, respectively, often within the first few weeks of treatment and frequently accompanied by significant weight gain and peripheral edema (Tian et al., 2025). Similar lipid disturbances have also been reported with newer targeted agents, including selpercatinib (Thein et al., 2021) and sotorasib (Skoulidis et al., 2021), supporting the need for systematic metabolic monitoring within oncologic treatment protocols.

The management of dyslipidemia is addressed in dedicated guidelines and includes statins, ezetimibe, bempedoic acid, and fibrates as first- and second-line treatment. More potent agents, such as PCSK9 inhibitors and inclisiran, are generally reserved for patients who fail to achieve lipid targets or have contraindications to first-line therapies (Mach et al., 2025b). Notably, despite substantial lipid elevations, both oncologic and cardiovascular outcomes were superior in patients treated with lorlatinib compared with crizotinib at 5 years. However, these findings should be interpreted with caution, as atherosclerosis progression and the development of clinical CV events typically follow a decadal latency. Moreover, patients who developed hyperlipidemia in clinical trials were managed using predefined protocols, including statins with minimal cytochrome–mediated drug–drug interactions (Shaw et al., 2020). Emerging real-world evidence suggests that CV risk in unselected patient populations may be more pronounced than that observed in strictly controlled trial settings (Lin et al., 2025). As survival in ALK-positive NSCLC continues to improve, the cumulative burden of therapy-induced hyperlipidemia may become a critical determinant of long-term outcomes. Consequently, lipid-lowering interventions will not be a secondary concern but a strategic intervention to prevent delayed CV adverse events.

### 7.4 Venous thromboembolism

Cancer-associated venous thromboembolism (VTE) remains a frequent and clinically relevant complication in NSCLC. Observational data suggest particularly high thrombotic rates in molecularly defined subsets regularly treated with targeted therapies, such as ALK- and ROS1-rearranged disease and the BsAb amivantamab, with many events occurring around the time of diagnosis or during early treatment phases. While the causal contribution of TKIs is difficult to disentangle from tumor biology, thrombotic risk should be considered dynamic and periodically reassessed during targeted treatment (Al-Samkari et al., 2020; Chiari et al., 2020). Hospitalized patients require thromboprophylaxis with low-dose anticoagulation according to their acute medical illness and mobility (Key et al., 2020). Ambulatory patients receiving systemic therapy should be individually evaluated for the VTE risk (Table 2). International guidelines recommend the use of validated risk assessment models, such as the Khorana score (Khorana et al., 2008) and COMPASS-CAT (Gerotziafas et al., 2017), to identify higher-risk candidates for prophylaxis discussions. However, there is limited discrimination of the Khorana score in lung cancer, and a low percentage of lung cancer patients in the COMPASS-CAT derivation cohort (Gerotziafas et al., 2017). Heterogeneous external validation results in NSCLC have been reported, even if the COMPASS-CAT demonstrated the better discriminative value in a small cohort of 118 outpatients with lung cancer (Rupa-Matysek et al., 2018). Several risk assessment models specific to lung cancer at different stages have been developed with varying performance results (Xiong et al., 2023). The Thrombo-NSCLC score is specifically proposed for NSCLC patients: it encompasses the measurement of factor VIII and soluble P-selectin, whose higher value predicts VTE event in a cohort of 90 patients with NSCLC (AUC 0.93) (Castellón Rubio et al., 2020).

**TABLE 2:** List of factors being considered during venous thromboembolism risk assessment in cancer patients.

Patient-related factors
Advanced age Comorbidities Female sex Hereditary coagulation defects: Factor V and factor II mutations Acquired coagulation defects Performance status Mobility Prior venous thromboembolism event
Cancer-related factors
Site Histology (adenocarcinoma) Stage (advanced, metastatic) Genetic characteristics (JAK2 or KRAS mutations) Time between diagnosis and treatment start
Treatment-related factors
Cancer therapy Central venous catheters Hospitalization Recent or planned major surgery

Thromboprophylaxis may be considered with apixaban, rivaroxaban, or low-molecular-weight heparin (LMWH), provided there are no significant risk factors for bleeding or drug interactions. Generally, prophylactic anticoagulation in advanced or metastatic lung cancer is not routinely suggested even if the individual patient’s bleeding risk profile is low (Farge et al., 2022): it does not confer an overall survival improvement (Key et al., 2020), even if some benefit has been reported in lung cancer (Liu et al., 2018), but mainly limited to SCLC (Altinbas et al., 2004; Yu et al., 2016). Prophylactic anticoagulation should be considered in case of treatment with amivantamab or amivantamab–lazertinib given the high rate of VTE during their treatment ranging from 10% to 37% for the latter combinations (VTE with Amivantamab plus Chemotherapy in NSCLC, 2024).

Guidelines recommendations endorse apixaban, edoxaban, or rivaroxaban as first-line options for the treatment of symptomatic or incidental VTE in patients with cancer without major bleeding-risk features or relevant drug–drug interactions. This is supported by randomized trials showing non-inferiority of direct activated factor X inhibitors versus dalteparin for recurrent VTE (Raskob et al., 2018; Young et al., 2018; Agnelli et al., 2020), with a trade-off of increased clinically relevant non-major bleeding for edoxaban and rivaroxaban, most pronounced in luminal gastrointestinal or genitourinary cancers, which is less typical of NSCLC (Sabatino et al., 2020).

LMWH remains preferred when bleeding risk is high, or in the presence of significant thrombocytopenia, gastrointestinal absorption concerns, advanced chronic kidney disease, or clinically meaningful interactions with anticancer agents involving CYP3A4 cytochrome and P-glycoprotein pathways (Patil et al., 2025). In thrombocytopenia, full-dose anticoagulation is generally supported when platelets are >50,000/µL; half-dose LMWH may be considered at 25,000–50,000/µL after multidisciplinary discussion, whereas <25,000/µL requires individualized decisions (Giustozzi et al., 2020; Lyon et al., 2022).

The minimum recommended duration of therapeutic anticoagulation is 6 months, with extension beyond 6 months advised when malignancy is active, metastatic, or anticancer therapy continues, balancing the overall risk of the patient, thrombotic and hemorrhagic. Extended LMWH strategies up to 12 months have shown feasibility in prospective cohorts (Francis et al., 2015). In case of recurrent VTE during anticoagulation, physicians must carefully assess adherence, disease progression, and interacting medications; switching strategy, typically from direct oral anticoagulant to LMWH, is indicated.

The role of inferior vena cava filters remains uncertain and controversial. Observational studies suggest the absence of overall survival in patients receiving an inferior vena cava filter with an absolute contraindication for anticoagulation (Brunson et al., 2017).

### 7.5 Heart failure

According to the baseline CV assessment, if LVEF is impaired or significant risk factors are present, referral to a cardio-oncology specialist for risk stratification is warranted to address all CV issues and optimize drug therapy.

Ongoing monitoring is essential for early detection of subclinical cardiac damage. Clinically, inquiring about HF symptoms, such as exertional dyspnea, nocturnal paroxysmal dyspnea, and edema, should be performed at each visit, since new-onset dyspnea may be mistakenly attributed solely to lung cancer rather than considering a cardiac contribution (Table 3). In patients receiving higher-risk regimens, periodic transthoracic echocardiography is recommended (Lyon et al., 2022). For example, in patients on Osimertinib, three-monthly echocardiography to screen for new LV dysfunction is suggested, given that the majority of LVEF declines occur within the first year. Similarly, in the case of VEGF inhibitors, serial echocardiographic evaluations are advised.

**TABLE 3:** Features and clinical management of cancer patients according to heart failure presentation and severity.

Symptoms	Grade	Features	Clinical management
Asymptomatic HF	Mild	LVEF≥50% Plus new relative decline in global longitudinal strain by >15% from baseline and/or new rise in cardiac troponin or natriuretic peptides	Narrow echocardiographic evaluation and biomarker measures Consider starting cardioprotective drugs, RASi, and beta-blockers
	Moderate	New LVEF reduction by ≥ 10 percentage points to a mildly reduced LVEF (40%–49%) OR new LVEF reduction by < 10 percentage points to mildly reduced LVEF (40%–49%) plus either a new relative decline in global longitudinal strain by >15% from baseline or a new rise in cardiac troponin or natriuretic peptides	Starting cardioprotective drugs, including RASi and beta-blockers, according to guidelines Continue targeted therapy and strict cardiovascular monitoring
	Severe	New LVEF reduction to <40%	Full therapy for HF with reduced LVEF. Targeted therapy interruption
Symptomatic HF	Mild	Mild HF symptoms	No intensification of therapy is required Multidisciplinary team to evaluate targeted therapy interruption vs. continuation
	Moderate	Moderate symptoms (NYHA II-III) not requiring hospitalization	Intensification of diuretic and HF therapy Interrupt targeted therapy. Multidisciplinary team to evaluate targeted therapy: definite interruption vs. reassumption
	Severe	HF hospitalization	Treatment of acute exacerbation of HF, including intravenous diuretic and non-invasive mechanical ventilation Interrupt targeted therapy. Multidisciplinary team to evaluate targeted therapy: definite interruption vs. reassumption
	Very severe	Advanced HF with cardiogenic shock presentation	Inotropic support, mechanical circulatory support, or consideration of transplantation. Interrupt targeted therapy. Multidisciplinary team to evaluate targeted therapy: definite interruption vs. reassumption

HF, heart failure; LVEF, left ventricular ejection fraction; NYHA, new york heart association; RASi, renin-angiotensin system inhibitors.

**FIGURE 5:**
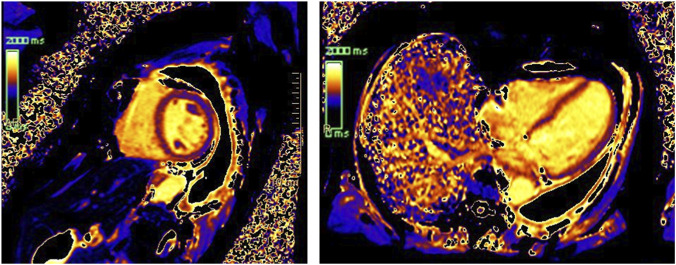
Cardiac magnetic resonance in a patient treated with an EGFR tyrosine kinase inhibitor. Native T1 mapping demonstrates increased myocardial T1 values, consistent with expansion of the extracellular volume and suggestive of early myocardial fibrotic remodeling. The short-axis view is shown on the left, and the four-chamber view on the right.

The global longitudinal strain represents a crucial parameter. It anticipates the drop in LVEF; moreover, a relative drop of >15% from baseline can signal incipient cardiotoxicity even if LVEF remains normal, although this threshold has been investigated in cancer patients treated with anthracyclines and trastuzumab encompassing different cancer types (Oikonomou et al., 2019). Cardiac magnetic resonance is emerging as a key modality for assessing myocardial injury during targeted therapies (Burrage and Ferreira, 2020). Through tissue characterization techniques, it can detect myocardial edema and fibrosis, potentially identifying subclinical damage before overt LV dysfunction develops (Figure 5). From the laboratory perspective, measuring natriuretic peptides and cardiac troponin at baseline and during therapy may help identify subclinical myocardial stress or injury in cancer patients (Hinrichs et al., 2020). Recommendations are largely based on expert consensus rather than robust randomized controlled clinical trials (Pudil et al., 2020), and most observational data come from cohorts of melanoma and breast cancer patients, highlighting a lack of specific data for NSCLC. Promising results from other biomarkers and microRNAs in breast cancer warrant further study, including in NSCLC patients (Gioffré et al., 2020).

When an asymptomatic LVEF drop or any HF symptoms occur, prompt intervention with guideline-directed medical therapy for HF is mandatory. There is general agreement in applying international guidelines for standard HF pharmacological therapy, also in cancer patients (McDonagh et al., 2021). Key points classically include a RASi or an angiotensin receptor-neprilysin inhibitor, a beta-blocker, a mineralocorticoid receptor antagonist, and a sodium-glucose cotransporter-2 inhibitor, up-titrated to tolerated target doses. Early initiation of these agents has been associated with improved LVEF recovery and outcomes in cancer therapy-related HF. Regular aerobic exercise can mitigate chemotherapy-related decline in LVEF and is likewise recommended in cancer patients who develop CTRCD, as long as their clinical status allows (Campia and Barac, 2016).

In NSCLC patients, special attention must be given to overlapping symptoms: dyspnea or fatigue might be attributed to cancer or anemia rather than HF, delaying care. Imaging can help clarify whether HF is the cause when the clinical presentation is ambiguous. Once HF is clinically diagnosed, outcomes in NSCLC patients are generally poor. Patients with lung cancer have a higher incidence of HF and major adverse CV events compared to patients with other cancer types (Mitchell et al., 2023). Furthermore, although HF admissions in lung cancer may be lower than those in other cancer types, the in-hospital mortality rate was the highest (Guha et al., 2019).

Deciding whether to continue, hold, or permanently stop a targeted cancer therapy after cardiotoxicity requires balancing oncologic necessity against cardiac risk, also considering the estimated mortality risk of HF and different cancer types (Stewart et al., 2010; Mitchell et al., 2023). A multidisciplinary team, including oncologists, cardiologists, and, if present, cardio-oncology specialists, should jointly evaluate each patient. Crucial issues concern the severity of HF and the reversibility of the LVEF reduction. The interruption of targeted therapy is often required to aofssess recovery in cardiac function, which is more likely when the diagnosis is made promptly (Cardinale et al., 2015). Evidence suggests that patients who develop HF with reduced LVEF with trastuzumab can improve and should be maintained on medical therapy for HF (Ewer et al., 2005). However, there is concern that cardiotoxicity may not always be reversible; thus, increased monitoring is recommended (Telli et al., 2007). Upon recovery, targeted therapy can be resumed with closer cardiac monitoring.

Rechallenge often involves a modified strategy: for instance, restarting at a reduced dose if feasible, or switching to a less cardiotoxic agent within the same class, according to different generations of TKIs. In case of persistent LVEF reduction, and no alternatives are available, targeted therapy resumption should be rigorously evaluated by a multidisciplinary team, also because no specific studies are available (Curigliano et al., 2020).

## 8 Future perspectives

The future of CV risk management in NSCLC is closely intertwined with the rapid evolution of therapeutic strategies. As molecularly targeted therapies, next-generation ADCs, and immune-engaging agents continue to expand across disease stages and treatment lines, CV toxicity is expected to emerge as an increasingly relevant determinant of treatment feasibility and long-term outcomes. Management of CV consequences cannot be postponed (Table 4).

**TABLE 4:** General suggestions for targeted therapy resumption after main cardiovascular toxicities (Herrmann et al., 2022; Lyon et al., 2022; Mitchell et al., 2023).

QTc prolongation
Targeted therapy continuation until QTc <500 msec Withdrawn therapy until QTc <481 msec. Check electrolytes Torsade de pointes/life-threatening ventricular arrhythmia: definite stop drug-class therapy
Bradycardia
Usually asymptomatic until 45 bpm Symptomatic (syncope or pre-syncope): Withdrawal of other drugs lowering heart rate (such as beta-blockers). Targeted therapy dosage reduction should be considered
Systemic arterial hypertension
If not emergency hypertension (with acute target-organ damage), introduce RASi, diuretics, beta blockers, and/or dihydropyridine calcium channel blockers until target values are achieved If uncontrolled hypertension: continuation of antihypertensive medications, and reduction of targeted therapy dose may be considered If emergency hypertension: definite withdrawal of the cancer therapy and treat hypertension, usually with intravenous medications. Shift to a different drug class
Dyslipidemia
Usually not requiring cancer-therapy modification, implement lipid-lowering drugs considering drug-drug interaction and life expectancy
LVEF reduction
Early treatment as soon as the first signals of impairment are appreciated If LVEF <40% or heart failure symptoms, withdraw targeted therapy, start or potentiate heart failure medications In case of LVEF recovery, at least 45%, continue heart failure medications, resumption of therapy with dose reduction, and closer cardiac monitoring In case of persistent LVEF reduction or symptomatic heart failure, it is suggested to switch to an alternative regimen or a different, less toxic drug of the same class
Venous thromboembolism
If not severe or life-threatening pulmonary embolism, treat the thrombotic event, consider long-term treatment according to the patient’s risk profile, and continue targeted therapy If severe or life-threatening pulmonary embolism, consider definite withdrawal of the ongoing targeted therapy and switch to another class
Peripheral edema
Generally not requiring withdrawal of cancer therapy Start diuretic therapy and compression stockings. Careful differential diagnosis with heart failure
Myocardial infarction or stroke (arterial thrombosis)
Generally, permanent discontinuation of the drug class

LVEF, left ventricular ejection fraction; RASi, renin-angiotensin system inhibitors.

In the coming years, the traditional distinction between short-term treatment-related adverse events and long-term CV sequelae will likely become less meaningful, particularly as NSCLC is progressively managed as a chronic condition in selected molecular subgroups.

One major future challenge will be the CV impact of emerging and increasingly potent drug platforms. Next-generation ADCs, characterized by higher drug-antibody ratios, membrane-permeable payloads, and enhanced bystander effects, are expected to achieve deeper and more durable tumor responses. However, these same features may increase off-target exposure of cardiac and vascular tissues, potentially unmasking novel or delayed CV toxicities that are not fully captured in clinical trials. Similarly, the broader development of BsAbs and other immune-engaging therapies may lead to more frequent and complex inflammatory CV phenotypes, particularly as these agents move beyond heavily pretreated populations.

Another key area of future development lies in the transition from class-based to mechanism-based CV risk assessment. Rather than focusing on individual drugs or molecular targets, future cardio-oncology strategies in NSCLC will likely prioritize shared biological pathways, including mitochondrial dysfunction, endothelial injury, inflammatory signaling, and metabolic remodeling. This shift may facilitate earlier identification of patients at risk for cumulative or delayed cardiotoxicity and support the development of targeted cardioprotective interventions applicable across multiple therapeutic classes.

An additional and increasingly relevant dimension of cardiovascular risk in NSCLC is the integration of locoregional treatments, particularly thoracic radiotherapy. Advances in radiation techniques, including intensity-modulated radiotherapy and proton therapy, have substantially reduced cardiac exposure; however, radiation-induced cardiovascular injury remains clinically relevant, especially in the context of mediastinal or left-sided irradiation. As radiotherapy is more frequently combined or sequenced with systemic targeted and immune-based therapies, the potential for synergistic or cumulative cardiovascular damage warrants careful consideration in future risk models.

Advances in precision cardio-oncology are also expected to reshape clinical practice. Baseline CV evaluation will remain essential, but future models are likely to incorporate dynamic risk stratification based on longitudinal biomarkers, advanced imaging modalities, and real-time clinical data. The integration of circulating biomarkers of myocardial injury and stress with functional imaging techniques, such as global longitudinal strain and cardiac magnetic resonance, may enable the detection of subclinical injury before irreversible dysfunction develops. In parallel, artificial intelligence-driven predictive models may offer opportunities to personalize monitoring intensity and preventive strategies, although prospective validation will be critical before widespread adoption.

From a clinical research perspective, future NSCLC trials will need to more systematically address CV safety. As therapies are increasingly combined and administered earlier in the disease course, traditional trial designs may underestimate long-term CV risk. Incorporating predefined CV endpoints, standardized toxicity definitions, and extended follow-up periods will be essential for accurately characterizing risk profiles. Importantly, future studies should also account for the sequencing of systemic therapies and radiotherapy, as different treatment trajectories may differentially modulate cumulative cardiovascular risk. Moreover, including patients with controlled CV comorbidities may improve the generalizability of trial findings and better reflect real-world populations.

Finally, future perspectives in NSCLC cardio-oncology should point toward a more integrated and anticipatory model of care. Rather than reacting to clinically overt CV events, future strategies need to emphasize early intervention and prevention, with CV considerations embedded into oncologic decision-making from the outset. This approach has the potential to preserve both cardiac function and treatment continuity, ultimately maximizing the benefits of increasingly effective anticancer therapies.

## Glossary

ACEi: angiotensin-converting enzyme inhibitor
ALK: anaplastic lymphoma kinase
AMPK: AMP-activated protein kinase
ARB: angiotensin receptor blockers
ADC: antibody-drug conjugate
BiTE: bispecific T-cell engager
BsAb: bispecific antibody
BP: blood pressure
CAR-T: chimeric antigen receptor T
CRS: cytokine release syndrome
CTRCD: cancer treatment-related cardiovascular disease
CV: cardiovascular
EGFR: epidermal growth factor receptor
FGFR: fibroblast growth factor receptor
HER2: human epidermal growth factor receptor 2
**I**_**Kr**_: rapid delayed rectifier potassium current
**I**_**Ks**_: slow delayed rectifier potassium current
LMWH: low-molecular-weight heparin
LV: left ventricle
LVEF: left ventricular ejection fraction
HF: heart failure
MAO: monoamine oxidase
MAPK/ERK: mitogen-activated protein kinase/extracellular signal-regulated kinase
MET: mesenchymal-epidermal transition
NOX4: NADPH oxidase 4
NO: nitric oxide
NSCLC: non-small cell lung cancer
NTRK: neurotrophic tyrosine receptor kinase
PDGFR: platelet-derived growth factor receptor
PI3K: phosphoinositide 3-kinase
RASi: renin-angiotensin system inhibitors
ROS: reactive oxygen species
T-DXd: Trastuzumab-deruxtecan
TKI: tyrosine kinase inhibitor
VEGF: vascular endothelial growth factor
VTE: venous thromboembolism
